# The novel LSD1 inhibitor ZY0511 suppresses diffuse large B-cell lymphoma proliferation by inducing apoptosis and autophagy

**DOI:** 10.1007/s12032-021-01572-0

**Published:** 2021-09-07

**Authors:** Huan Liu, Jing Wei, Na Sang, Xi Zhong, Xia Zhou, Xinyu Yang, Jing Zhang, Zeping Zuo, Yang Zhou, Shengyong Yang, Junrong Du, Yinglan Zhao

**Affiliations:** 1 Department of Pharmacology, Key Laboratory of Drug Targeting and Drug Delivery System of the Education Ministry, Sichuan Engineering Laboratory for Plant-Sourced Drug and Sichuan Research Center for Drug Precision Industrial Technology, West China School of Pharmacy, Sichuan University, Chengdu, 610041 China; 2 State Key Laboratory of Biotherapy and Cancer Center, West China Hospital, West China Medical School, and Collaborative Innovation Center for Biotherapy, Sichuan University, Chengdu, 610041 China

**Keywords:** LSD1 inhibitor, ZY0511, Diffuse large B-cell lymphoma, Apoptosis, Autophagy

## Abstract

Lysine-specific demethylase 1 (LSD1, also known as KDM1A) is an attractive agent for treatment of cancer. However, the anti-tumor effect of LSD1 inhibitors against diffuse large B-cell lymphoma (DLBCL) and the underlying mechanism are still unclear. Here, we report that *KDM1A* is overexpressed in human DLBCL tissues and negatively related to overall survival rate of DLBCL patients. ZY0511, a novel and potent LSD1 inhibitor developed by our group, inhibited the proliferation of human DLBCL cells. ZY0511 interacted with LSD1, induced methylation level of histone 3 lysine 4 and histone 3 lysine 9 in DLBCL cells. Mechanistically, transcriptome sequencing results indicated that ZY0511 induced the genes enrichment significantly related to cell cycle, autophagy, and apoptosis signaling pathways. Further study confirmed that ZY0511 blocked cell cycle at G0/G1 phase and expression of CDK4 and cyclin D1. ZY0511 decreased mitochondrial membrane potential and induced apoptosis, which can be reverted by a pan-caspase inhibitor, Z-VAD-FMK. Moreover, ZY0511 treatment significantly increased autophagy-associated marker proteins and autophagosomes formation in DLBCL cells. In vivo xenograft experiments confirmed that intraperitoneal administration of ZY0511 significantly suppressed SU-DHL-6 xenograft tumor growth in vivo. In conclusion, our findings identify that ZY0511 inhibits DLBCL growth both in vitro and in vivo via the induction of apoptosis and autophagy, and LSD1 inhibitor might be a promising strategy for treating DLBCL.

**Supplementary Information:**

The online version contains supplementary material available at 10.1007/s12032-021-01572-0.

## Introduction

Diffuse large B-cell lymphoma (DLBCL) is the most aggressive class of non-Hodgkin lymphomas (NHLs) [1]. The standard treatment strategy of rituximab in combination with a cocktail of chemotherapy agents comprising of cyclophosphamide, doxorubicin, prednisone, and vincristine (R-CHOP) extend the overall survival time of DLBCL patients [2]. However, there are still 40% of patients who do not have response to treatment or relapse after treatment [3], which made the outcome of DLBCL patients remain far from satisfactory. Therefore, it is urgent to develop novel drugs that can be effectively used for treatment of DLBCL patients.

Epigenetic dysregulation plays a crucial role in the initiation and development in human DLBCL [1, 4]. In 2004, Shi’s group identified that lysine-specific histone demethylase 1 (LSD1) which is encoded by *KDM1A* is an epigenetic regulatory factor [5]. LSD1 represses transcription via specially demethylating lysine residues of histone 3 at lysine 4 (H3K4) and activates transcription though specially demethylating lysine residues of histone 3 at lysine 9 (H3K9), respectively, thereby regulating the expression of target genes [6]. LSD1 is ubiquitously overexpressed in numerous cancers, including acute myeloid leukemia (AML), breast cancer, prostate cancer, neuroblastoma, and small cell lung cancer (SCLC), and its overexpression associates with the initiation and progression of malignant tumors and the overall survival time of patients [7, 8]. Therefore, LSD1 has attached much attention, and the biological function of LSD1 in hematological malignancies especially AML has been extensively studied. In AML, LSD1 interacts with ectopic Snail Family Transcriptional Repressor 1 (SNAI1) to induce myeloid development defects [9]. Furthermore, there are several studies suggested that LSD1 participates in lymphomagenesis and the progression of lymphoma. LSD1 is found to be essential for germinal centers (GC) formation and humoral immune response by interacting with B-Cell Lymphoma 6 Protein (BCL6) in lymphoma cells [6]. Conditional deletion of LSD1 obviously delayed BCL6-driven lymphomagenesis [10]. Above studies revealed that LSD1 play a crucial role in lymphoma, and it is feasible to treat DLBCL with LSD1 inhibitors.

In the last decades, numerous LSD1 inhibitors, including TCP, ORY-1001, ORY-2001, CC-90011, INCB059872, and IMG-7289 are undergoing clinical trials for treatment of AML, myelodysplastic syndromes (MDS), myeloproliferative neoplasms, SCLC, relapsed Ewing sarcoma, myelofibrosis, essential thrombocythemia, multiple sclerosis, mild to moderate Alzheimer’s disease [11]. Although the effect of LSD1 inhibitors in above diseases is under investigating, the effect of LSD1 inhibitors in DLBCL treatment remains largely unclear. Until now, there is only one LSD1 inhibitor, CC-90011, undergoing clinical trials for NHL therapy (Clinical trials identifier NCT02875223), and the preclinical data such as efficiency and safety of CC-90011 is unrevealed [9]. Thus, there is an urgent clinical need to develop LSD1 inhibitors for treating DLBCL.

In 2016, our group developed a potent LSD1 inhibitor, ZY0511, which potently and selectively inhibited LSD1 activity with an IC_50_ value of 1.7 nM [12]. Here, we aimed to clarify the anti-tumor effect and underlying mechanism of ZY0511 against DLBCL. We found that *KDM1A* was highly expressed in human DLBCL tissues and associated with the poor survival of DLBCL patients. By interacting with LSD1 in DCBCL cells, ZY0511 inhibited cell proliferation both in vitro and in vivo. Mechanistically, ZY0511 blocked cell cycle at G0/G1 phase, induced apoptosis, and induced autophagy via inactivation of mTOR/p70S6K signaling pathways. Our findings reveal that ZY0511 might be a promising treatment strategy for treating DLBCL.

## Materials and methods

### Antibodies and reagents

The main antibody information was listed below: LSD1 (CST, USA), H3 (CST, USA), H3K4me (CST, USA), H3K4me2 (CST, USA), H3K9me (CST, USA), H3K9me2 (CST, USA), phospho-p53 (Ser 15)(CST, USA), phospho-mTOR (Ser 2448)(CST, USA), p70 S6K (CST, USA), phospho-p70 S6k (Thr 421/Ser 424)(CST, USA), phospho-S6 (Ser235/236)(CST, USA), Beclin-1 (CST, USA), Bax (CST, USA), Cleaved Caspase-3 (CST, USA), GAPDH (CST, USA), Ki67 (Abcam, USA), mTOR (ZEN BIO, China), p53 (Proteintech, USA), Caspase-8 (Proteintech, USA), CDK4 (Proteintech, USA), CDK6 (Proteintech, USA), Cyclin D1 (Proteintech, USA), LC3B (Santa Cruz, Bolivia), p62/SQSTM1 (HUA BIO, China), S6 (Abclonel, China), PCNA(Gservice, China).

GSK2879552, 3MA, chloroquine (CQ), and Z-VAD-FAM were purchased from Selleckchem (Selleckchem, USA). ZY0511 was synthesized in laboratory as described previously [12].

### Cell lines and cell culture

The human DLBCL cell lines including SU-DHL-4, SU-DHL-6, SU-DHL-10, and Farage were purchased from American Type Culture Collection (ATCC, USA). The DLBCL cells were cultured in RPMI 1640 medium containing 20% fetal bovine serum (Gibco, Australia) at 37 °C and 5% CO_2_.

### Western blot

The human DLBCL cells were lysed with RIPA lysis buffer containing protease inhibitor and phosphatase inhibitors. Next, the bicinchoninic acid (BCA) method was used to quantify the protein concentrations. Proteins were isolated by SDS-PAGE gel, and then transferred onto polyvinylidene fluoride membranes (Millipore, USA). After blocking with 5% skimmed milk, the membranes were incubated with the specific antibodies at 4 °C overnight. The membranes were washed with TBST buffer three times and incubated with horseradish peroxidase (HRP) conjugated secondary antibodies at room temperature for 1 h. At last, the protein levels were visualized using Chemiluminescent HRP Substrate (Millipore, USA) and signals were detected using chemiluminescence imaging system.

### Cellular thermal shift assay (CETSA)

The DLBCL cells were seeded (2 × 10^5^ cells/mL) and treated with DMSO or ZY0511 at the final concentration of 200 μM at 37 °C for 1 h. The cells were harvested and then washed with pre-chilled PBS for twice, followed by resuspension in pre-chilled PBS containing protease inhibitor. The cell suspension was aliquoted into PCR tubes (1.5 × 10^6^ cells/tube) and heated at different temperatures (42 °C, 44 °C, 46 °C, 48 °C, 50 °C, 52 °C, 54 °C) for 3 min, followed by placed at room temperature for 3 min. Next, the sample was subjected to three freeze (in liquid nitrogen)—thaw cycles (at room temperature), and the supernatant was obtained by centrifugation at 20,000×*g* for 20 min at 4 °C. Subsequently, loading buffer was added, and then proteins were denatured at 100 °C for 10 min. Finally, the level of LSD1 was detected by western blot assay. Western blot signals based on densitometry method were quantified by Fiji and CETSA curves in intact cells were graphed by GraphPad Prism 8 software (GraphPad, USA).

### MTT assay

An MTT (Sigma, USA) experiment was conducted to assess the proliferation inhibitory rate of ZY0511 against DLBCL cells. The DLBCL cells were treated with various concentrations of ZY0511 in 96-well plates for 24 h (8 × 10^4^ cells/well), 48 h (4 × 10^4^ cells/well), 72 h (2 × 10^4^ cells/well), and 96 h (1 × 10^4^ cells/well), respectively. Then, 20 μL MTT (5 mg/mL) was added to form formazan. Optical density was measured using a microplate reader (Thermo Fisher Scientific, USA). The IC_50_ values were calculated by GraphPad Prism 8 software using XY modeling.

### EdU incorporation assay

The DLBCL cells were seeded (2 × 10^5^ cells/mL) and treated with ZY0511 at the final concentrations of 0.5, 1, 2 μM for 24 h. The DLBCL cells were grown in medium containing EdU, which is a thymidine analogue labeled cells in the proliferation phase for 2 h. Then, the EdU positive cells were stained with Cell-Light EdU Apollo488 In Vitro Flow Cytometry Kit according to Ribobo’s instructions (Ribobo, China). Cells were measured by flow cytometry (Agilent NovoCyte, USA) and analyzed using NovoExpress software (Agilent NovoCyte, USA).

### Real-time quantitative PCR (qRT-PCR)

Total RNA was isolated from cultivated DLBCL cells using AxyPrep™ Multisource Total RNA Miniprep Kit (Axygen, USA). PrimeScript™ RT reagent Kit with Gdna Eraser (TakaRa, Japan) was used to synthesize cDNA. RNA quality was evaluated by Real-Time system (Bio-Rad, USA) using the SYBR® Green Supermix (Bio-Rad, USA). Primers were listed in Table S1.

### RNA sequencing (RNA-seq)

RNA-seq experiment was conducted using a profiler service provided by Novogene Co. Ltd (Novogene, China). After 48 h of treatment with DMSO or ZY0511 (1 μM), total RNA was purified from SU-DHL-4 cells and stored in TRIzol (Invitrogen, USA). A fold-change > 1.5, FDR (false discovery rate) < 0.05, and *P* < 0.05 (Student’s t-test) were used to filter significant probe sets for each biological sample (n = 3). The gene counts of each transcript were calculated and DESeq2 was used for normalization to determine differentially expressed genes.

### Cell cycle analysis

The DLBCL cells were seeded (2 × 10^5^ cells/mL) and treated with ZY0511 at the final concentrations of 0.5, 1, 2 μM for 24 h. Then, the DLBCL cells were collected and fixed with 70% ethanol solution at 4 °C overnight. Fixed cells were stained with propidium iodide according to KeyGEN BioTECH’s instructions (KeyGEN BioTECH, China). Cells were measured by flow cytometry (Agilent NovoCyte, USA) [13] and analyzed using NovoExpress software (Agilent NovoCyte, USA).

### Apoptosis analysis

The DLBCL cells were seeded (2 × 10^5^ cells/mL) and treated with ZY0511 at the final concentrations of 0.5, 1, 2 μM for 48 h. Then, the DLBCL cells were harvested and stained with Annexin V-PE and 7-AAD according to BD’s instructions (BD, USA), followed by apoptosis analysis by flow cytometry [14].

### Mitochondrial membrane potential (ΔΨm) assay

The DLBCL cells were seeded (2 × 10^5^ cells/mL) and treated with ZY0511 at the final concentrations of 0.5, 1, 2 μM for 48 h. Then, the DLBCL cells were harvested and incubated with tetraethyl benzimidazolyl carbocyanine iodide (JC-1) according to Beyotime’s instructions (Beyotime, China), followed by ΔΨm assay by flow cytometry analysis.

### Cell number and viability assay determined by acridine orange/propidium iodide (AO/PI)

The DLBCL cells were seeded (2 × 10^5^ cells/mL) and treated with ZY0511 (2 μM) and/or Z-VAD-FMK (50 μM) for 48 h. After resuspending the DLBCL cells, 20 μL cell suspension was added 20 μL mixture solution of AO which stains living cells to green and PI which stains dead cells to red. A cell counter was used to determine the cell number and the percent of live cells and dead cells.

### Immunofluorescence (IF)

After ZY0511 treatment, DLBCL cells were harvested and fixed with 4% paraformaldehyde at 4℃ for 15 min. The cell suspension was dripped onto a glass slide and placed at 37 °C to dry the water. At room temperature, the DLBCL cells were permeabilized with 0.2% Triton X-100 solution for 20 min and the permeabilized cells were blocked with PBST containing 0.2% bovine serum albumin (BSA) for 1 h. Then, slides were incubated with primary antibody overnight at 4 °C, followed by incubating with Cy3 or FITC-conjugated secondary antibody for 1 h at room temperature with shaking [15]. Nuclei were labeled with DAPI. DeltaVision Ultra-high resolution microscope (GE Healthcare, USA) was used to analyze the slides using a 63 × NA oil objective.

### Transmission electron microscopy (TEM)

The DLBCL cells were seeded (2 × 10^5^ cells/mL) and treated with ZY0511 at the final concentration of 2 μM for 48 h. The treated cells were fixed with 2.5% glutaraldehyde. Then, cells were embedded in epoxy resin and imaged by JEM-1400 Plus TEM (JEOL, Japan) following standard TEM procedures.

### Subcutaneous xenograft models

NOD/SCID mice aged 5- to 6-week were purchased from the GemPharmatech Co., Ltd (GemPharmatech, China) and fed under specific pathogen-free (SPF) barriers. The SU-DHL-6 Cells (2 × 10^6^ cells/mouse/100 μL) were subcutaneously injected in the right flank of the mice. The tumor-bearing mice were randomly divided into three groups (*n* = 6 per group) when tumor volume reached 80–100 mm^3^. ZY0511 was suspended in 6% PEG4000, 2.4% Tween-20, and 91.6% ultrapure water, followed by nanocrystalline suspension preparation by high-pressure homogenization. ZY0511 (50 mg/kg and 100 mg/kg) or solvent were administrated once daily by intraperitoneal injection for 21 days. Tumor volumes and body weights were monitored every three days. Calculation formula: *V* (mm^3^) = (*a* × *b*^2^)/2 (*V* is the tumor volume, *a* is the length, and *b* is the width). At the end of the experiment on day 21, the tumor tissues were stripped and fixed in 4% paraformaldehyde for further experiments.

### Immunohistochemistry analysis and hematoxylin and eosin (H&E) staining

After treating with ZY0511 for 21 days, tumor tissues were collected from mice bearing SU-DHL-6 tumors. After being fixed in 4% paraformaldehyde for 48 h, the tumor tissues were embedded in paraffin, and then stained with Ki67 and PCNA by immunohistochemistry analysis. H&E staining tissue samples were also performed.

### Statistical analysis

Data are presented as the means ± SEM. GraphPad Prism 8 software was used to perform statistical analyses. The statistical significance of the data between two experimental groups was detected by a two-tailed Student's *t*-test. The statistical significance of the data among multiple groups was tested with one-way ANOVA. The survival analysis was performed by the log-rank test. *P < 0.05, **P < 0.01, and ***P < 0.001 were considered statistically significant. All in vitro experiments were performed for three times.

## Results

### *KDM1A* is overexpressed in human DLBCL tissues and negatively related to overall survival of patients

**Fig. 1:**
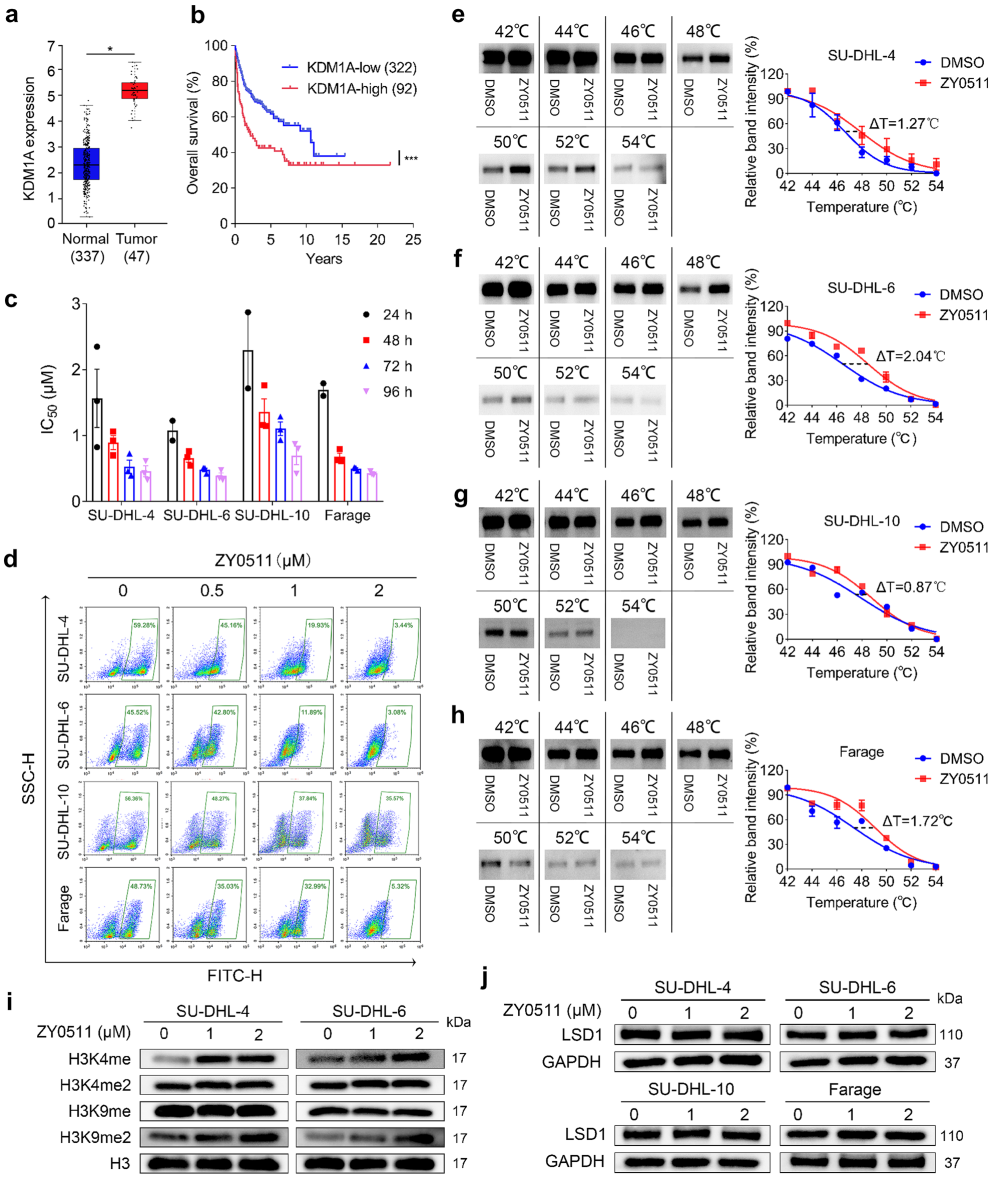
ZY0511 inhibits DLBCL cells proliferation and interacts with LSD1 in DLBCL Cells. **a** The mRNA expression of *KDM1A* in human DLBCL tissues and normal lymph node tissues from GEPIA web (*P < 0.05; student’s t-test). **b** Kaplan–Meier survival analysis was used to analyze the relationship between overall survival rates of DLBCL patients and the mRNA expression of *KDM1A* (***P < 0.001; log-rank test). **c** The DLBCL cells proliferation after various concentration of ZY0511 treatment for 24–96 h was detected by MTT assay (*n* = 3). GraphPad Prism 8 was used to calculate IC_50_ values. **d** The anti-proliferation effect of ZY0511 against SU-DHL-4, SU-DHL-6, SU-DHL-10, and Farage by EdU incorporation assay (*n* = 3). **e–h** CETSA melt curve from 42 to 54 °C of DLBCL cells lysates with or without ZY0511 incubation. The representative graphs (left) and quantitation (right) of western blot results. The data are presented as the means ± SEM from three independent experiments. **i**–**j** Western blot detection of H3K4me, H3K4me2, H3K9me, and H3K9me2 (**i**) and LSD1 (**j**) after ZY0511 treatment (1, 2 μM) (*n* = 3). Histone 3 (H3) is used as nucleus reference protein and GAPDH was used as the reference protein

To explore the clinical significance and correlation of LSD1 in DLBCL patients, we used a database containing 384 individual DLBCL tissues from the GEPIA2 website (http://gepia2.cancer-pku.cn/#index) to analyze the expression of *KDM1A* mRNA in human DLBCL tissues and normal lymph nodes. The results showed that high expression of *KDM1A* was observed in human DLBCL tissues compared with that in normal lymph nodes (Fig. 1a). Kaplan–Meier survival analysis revealed that the *KDM1A* low expression group (*KDM1A*-low) had a higher survival rate than that of the *KDM1A* high expression group (*KDM1A*-high) based on the data from Genomicscape website (http://www.genomicscape.com/), suggesting that high *KDM1A* expression in DLBCL tissues was associated with poor prognosis of patients (Fig. 1b). In short, these data indicate that the high expression of *KDM1A* in human DLBCL tissues is tightly correlated with the progression of DLBCL.

### ZY0511 inhibits the proliferation of DLBCL cells

To study the potential functional roles of ZY0511 in DLBCL cells, the cell proliferation after ZY0511 treatment was investigated by the MTT assay. The results revealed that ZY0511 time dependently displayed considerable anti-viability activities against SU-DHL-4, SU-DHL-6, SU-DHL-10, and Farage cells with IC_50_ values ranging from 0.33 to 2.87 μM (Fig. 1c). Both SU-DHL-4 and SU-DHL-6 cells were sensitive to ZY0511. Response to ZY0511, the IC_50_ values of SU-DHL-4 and SU-DHL-6 were 1.57 and 1.08 μM at 24 h, 0.89 and 0.66 μM at 48 h, 0.53 and 0.48 μM at 72 h, 0.46 and 0.39 μM at 96 h, respectively.

EdU incorporation assay further confirmed that, compared with the control group, ZY0511 treatment decreased the EdU positive cells from 61.61 to 10.25% in SU-DHL-4 cells, from 47.00 to 9.30% in SU-DHL-6 cells, from 54.18 to 47.40% in SU-DHL-10 cells, and from 52.76 to 12.23% in Farage cells. Above results indicated that ZY0511 obviously decreased the DNA synthesis of DLBCL cells in a concentration-dependent manner, which confirmed the cell proliferation inhibition induced by ZY0511 (Fig. 1d).

### ZY0511 interacts with LSD1 in DLBCL cells

We performed CETSA to evaluate whether ZY0511 interacted with LSD1 in DLBCL cells [16]. The results revealed ZY0511 (100 μM) treatment induced large thermal shifts of LSD1 in DLBCL cells compared with control. ZY0511 treatment increased the melt temperature of LSD1 in SU-DHL-4, SU-DHL-6, SU-DHL-10, and Farage cells 1.27, 2.04, 0.87, 1.72 °C, respectively (Fig. 1e–h). These results suggested that ZY0511 interacted with LSD1, thus increased the thermal stability of LSD1 in DLBCL cells, which is a crucial factor in the treatment effect.

To further confirm ZY0511 interacted with LSD1 and affected its catalytic function in DLBCL cells, we detected the histone methylation level as LSD1 demethylates lysine residues of histone H3K4 and H3K9. As SU-DHL-4 cells and SU-DHL-6 were most sensitive to ZY0511 treatment, they were chosen for further investigation. The result demonstrated that ZY0511 significantly increased the H3K4me and H3K9me2 levels in DLBCL cells (Fig. 1i). Simultaneously, ZY0511 had no influence on expression of LSD1 (Fig. 1j). Taken together, ZY0511 interacted with LSD1 in DLBCL cells, conforming that ZY0511 targeted LSD1 and exhibited its catalytic function.

### ZY0511 induced gene expression changes in cell cycle, apoptosis, and autophagy pathways

**Fig. 2:**
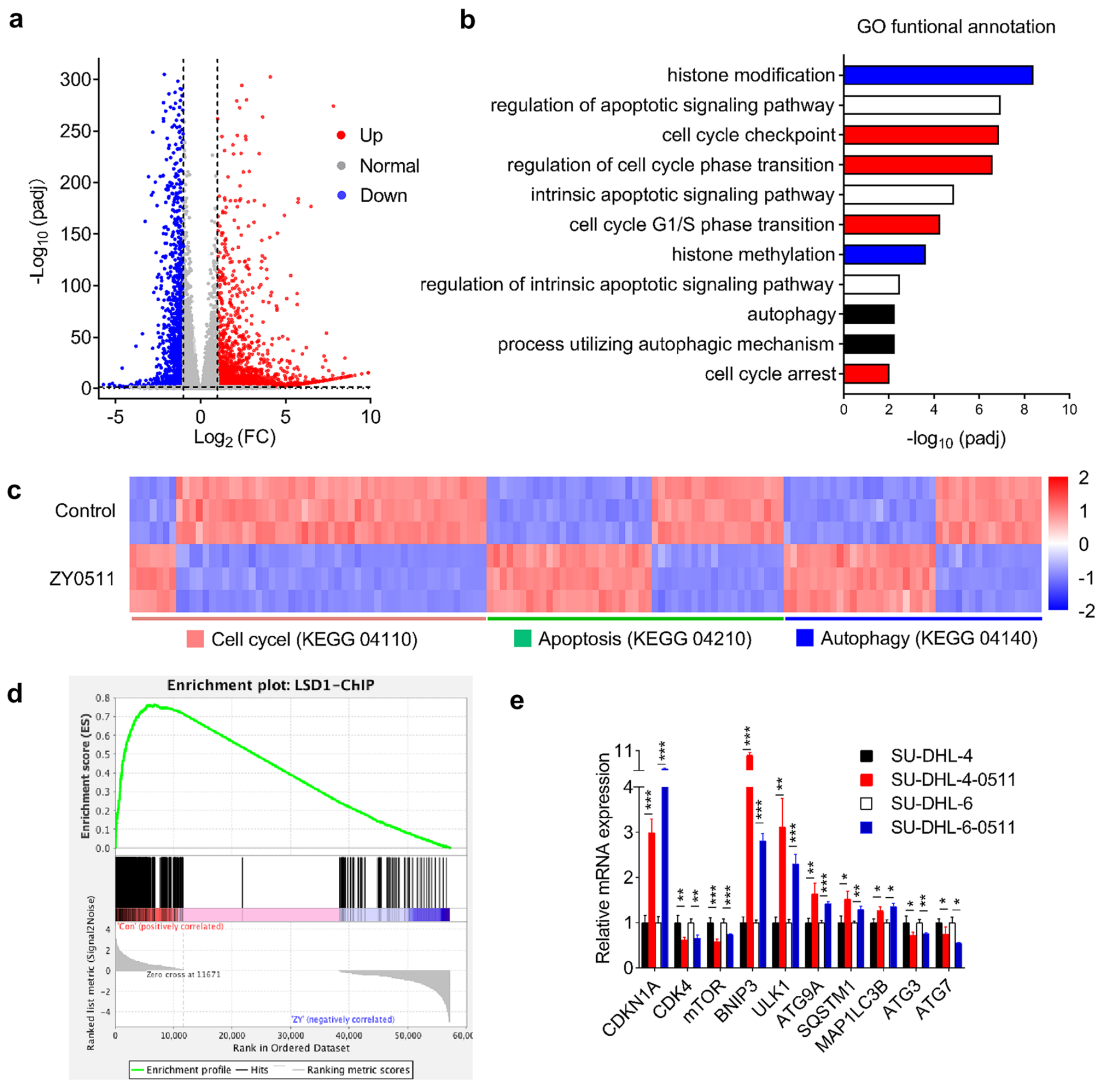
ZY0511 induces gene expression changes in DLBCL cells. **a** Volcano plot of differentially expressed genes in SU-DHL-4 cells after ZY0511 treatment (from RNA-seq data). Red dots represent significantly up-regulated genes, blue dots represent significantly down-regulated genes, and gray dots represent no meaningful changed genes. **b** Representative GO term analysis of significantly changed genes after ZY0511 treatment. **c** Heatmaps for the expression patterns of differentially expressed genes (adjusted *P* < 0.05), in cell cycle, apoptosis, and autophagy gene sets. **d** GSEA plots show enrichment of gene sets regulated following treatment of SU-DHL-4 cells with 1 μM ZY0511. **e** qRT-PCR validation of expression changes of 10 genes selected from RNA-seq in DLBCL cells. The data are presented as the mean ± SEM from three independent experiments, *P < 0.05, **P < 0.01, ***P < 0.001, student’s *t*-test

RNA-seq technology was applied to investigate the genes and signal pathways that mediate the antitumor effect of ZY0511. RNA-seq results showed that ZY0511 treatment obviously disturbed gene expression of SU-DHL-4 cells (Fig. 2a). Gene ontology (GO) enrichment analysis of the differentially expressed genes revealed enrichment for transcriptional programs significantly related to cell cycle, autophagy, and apoptosis signaling pathways (Fig. 2b), which were further confirmed by KEGG pathway analysis (Fig. 2c). The downregulated and upregulated representative gene lists of the RNA-seq analysis listed in Table S2. Among cell cycle-related genes, ZY0511 up-regulated *CDKN1A* expression by 5.6 folds and down-regulated *CDK4* expression by 60%. Among apoptosis and autophagy-related genes, ZY0511 increased *BNIP3* expression by 7.3 folds, *ULK1* expression by 3.6 folds, *ATG9A* expression by 2.0 folds, *SQSTM1* expression by 1.9 folds, *MAP1LC3B* expression by 2.0 folds, *SESN2* expression by 1.4 folds, and decreased *mTOR* expression by 50%, *ATG3* expression by 60%, *ATG7* expression by 10%. Using LSD1 downstream gene sets (http://cistrome.org/CistromeCancer/) for gene set enrichment analysis (GSEA), it was found that ZY0511 treatment induced the change of a subset of LSD1 downstream transcriptional programs in SU-DHL-4 (Fig. 2d), suggesting that ZY0511 changes numerous genes regulated by LSD1.

We next performed qRT-PCR analysis to verify above-mentioned representative differential genes selected from RNA-seq (Fig. 2e). Validation results were consistent with RNA-seq findings. Collectively, the anti-tumor effect of ZY0511 may be associated with cell cycle arrest, apoptosis, and autophagy pathways.

### ZY0511 induced DLBCL cell cycle arrest at G0/G1 phase

**Fig. 3:**
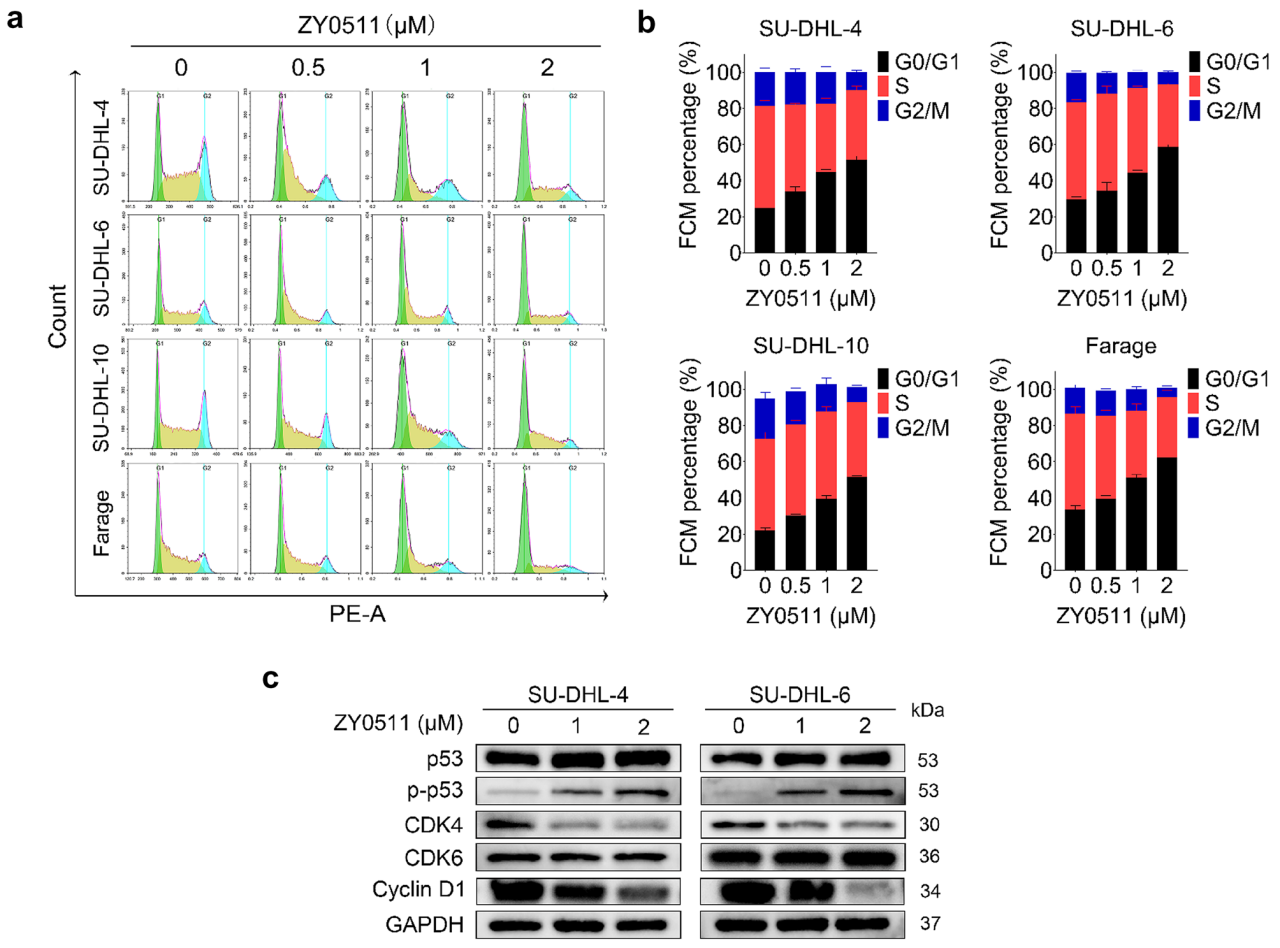
ZY0511 induces cell cycle arrest at G0/G1 phase in DLBCL cells. **a**, **b** Cell cycle analysis of SU-DHL-4, SU-DHL-6, SU-DHL-10, and Farage after ZY0511 treatment. The data are presented as the means ± SEM from at least three independent experiments. **c** Western blot detection of cell cycle pathway proteins (*n* = 3)

The effect of ZY0511 on the cell cycle was assessed in DLBCL cell lines. After ZY0511 treatment, the proportion of cells in the G0/G1 phase in SU-DHL-4, SU-DHL-6, SU-DHL-10, and Farage cells increased from 24.73 to 51.45%, from 29.49 to 58.75% %, from 22.29 to 51.54%, and from 33.73 to 62.44%, respectively. Above results revealed that ZY0511 concentration dependently induced cell cycle arrest at G0/G1 phase (Fig. 3a, b).

As expected, immunoblot analyses found the levels of p53 and p-p53 was elevated, CDK4 and Cyclin D1 was reduced, whereas the expression of CDK6 did not change significantly (Fig. 3c). The above data indicated that ZY0511 induces G0/G1 cell cycle phase arrest in vitro.

### ZY0511 exhibits anti-tumor effects by inducing apoptosis

**Fig. 4:**
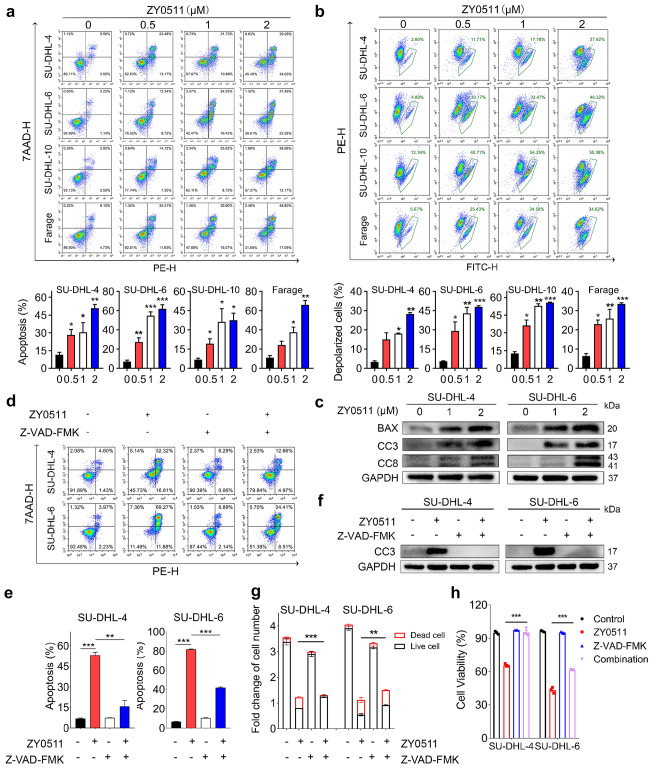
ZY0511 induces apoptosis in DLBCL Cells. **a** Flow cytometric histograms of apoptotic DLBCL cells after ZY0511 treatment at the indicated concentrations. The data are presented as the means ± SEM from three independent experiments. *P < 0.05, **P < 0.01, ***P < 0.001, one-way ANOVA followed by Dunnett’s test. **b** ΔΨm assay in DLBCL cells after ZY0511 treatment by flow cytometry. The data are presented as the means ± SEM from three independent experiments. *P < 0.05, **P < 0.01, ***P < 0.001, one-way ANOVA followed by Dunnett’s test. **c** Western blot detection of BAX, CC3, and CC8 in DLBCL cells after ZY0511 treatment (*n* = 3). **d**, **e** Flow cytometric histograms of apoptotic DLBCL cells after ZY0511 and Z-VAD-FMK cotreatment. The data are presented as the means ± SEM from three independent experiments. *P < 0.05, **P < 0.01, ***P < 0.001, one-way ANOVA followed by Dunnett’s test. **f** Western blot detection of CC3 after ZY0511 and Z-VAD-FMK treatment (*n* = 3). **g**, **h** The fold change of cell number and cell viability of DLBCL cells after the combination treatment of ZY0511 and Z-VAD-FMK by AO/PI assay. The data are presented as the means ± SEM from three independent experiments. *P < 0.05, **P < 0.01, ***P < 0.001, one-way ANOVA followed by Dunnett’s test

To study whether ZY0511 induced apoptosis in DLBCL, we performed flow cytometry analysis to detect apoptosis cells using Annexin V/7AAD staining. After ZY0511 treatment, the proportion of apoptotic cells in SU-DHL-4, SU-DHL-6, SU-DHL-10, and Farage cells increased from 11.30 to 50.77%, from 5.61 to 65.52%, from 6.78 to 37.66%, and from 10.95 to 66.05%, respectively. Results indicated that apoptosis was concentration dependently induced in ZY0511 group compared with the control (Fig. 4a). Loss of mitochondrial membrane potential (ΔΨm) was also associated with apoptosis. Our results showed that ZY0511 treatment increased the proportion of depolarized cells, which represents the ΔΨm alteration, in SU-DHL-4, SU-DHL-6, SU-DHL-10, and Farage cells from 3.24 to 28.29%, from 5.57 to 47.99%, from 12.54 to 55.65%, and from 10.95 to 66.05%, respectively (Fig. 4b), suggesting that ZY0511 treatment decreased the ΔΨm of DLBCL cells. Moreover, western blot analysis results showed that ZY0511 treatment increased the expression of cleaved caspase-3 (CC3) which is an indicator of cellular apoptosis, and Bcl-2-associated X protein (Bax) which is a pro-apoptotic protein involved in the mitochondrial-mediated endogenous apoptosis (Fig. 4c). Furthermore, ZY0511 induced expression of cleaved caspase-8 (CC8), suggesting activation of extrinsic apoptosis pathway (Fig. 4c). Collectively, above findings suggested that ZY0511 induces both endogenous and exogenous apoptosis [17, 18].

Then, to determine whether apoptosis is important for DLBCL cells death induced by ZY0511, we added Z-VAD-FMK, a pan-caspase inhibitor, to culture cells and detect apoptosis cells using Annexin V/7AAD staining. ZY0511 increased the number of apoptotic SU-DHL-4 cells by 7.9 folds (from 6.74 to 53.13%), and Z-VAD-FMK cotreatment reduced apoptotic SU-DHL-4 cells by about 70% (from 53.13 to 15.72%). ZY0511 increased the number of apoptotic SU-DHL-6 cells by 12.4 folds (from 6.62 to 81.78%), and Z-VAD-FMK cotreatment reduced apoptotic SU-DHL-6 cells by about 49% (from 81.78 to 41.8%) (Fig. 4d, e). Western blot results further indicated that the expression of CC3 induced by ZY0511 was completely reverted by ZVAD-FMK (Fig. 4f). These results indicated that the apoptosis induced by ZY0511 was greatly reversed by ZVAD-FMK cotreatment.

Moreover, the effect of ZVAD-FMK on ZY0511 induced cell number and viability change were evaluated by AO/PI method. In SU-DHL-4 cells, Z-VAD-FMK cotreatment increased the number of live cells by about 56% (from 1.60 × 10^5^ to 2.51 × 10^5^ cells/mL) compared with ZY0511 group. In SU-DHL-6 cells, Z-VAD-FMK cotreatment increased the number of live cells by about 53% (from 1.13 × 10^5^ to 1.73 × 10^5^ cells/mL) compared with ZY0511 group (Fig. 4g). Compared with ZY0511 group, Z-VAD-FMK cotreatment elevated the cell viability from 66 to 95% in SU-DHL-4 cells and from 43 to 62% in SU-DHL-6 cells (Fig. 4h). These findings demonstrated that the cell viability decreased by ZY0511 treatment was attenuated after ZVAD-FMK treatment. Collectively, apoptosis is a very important mechanism involved in DLBCL cells death induced by ZY0511.

### ZY0511 exhibits anti-tumor effects by inducing autophagy

**Fig. 5:**
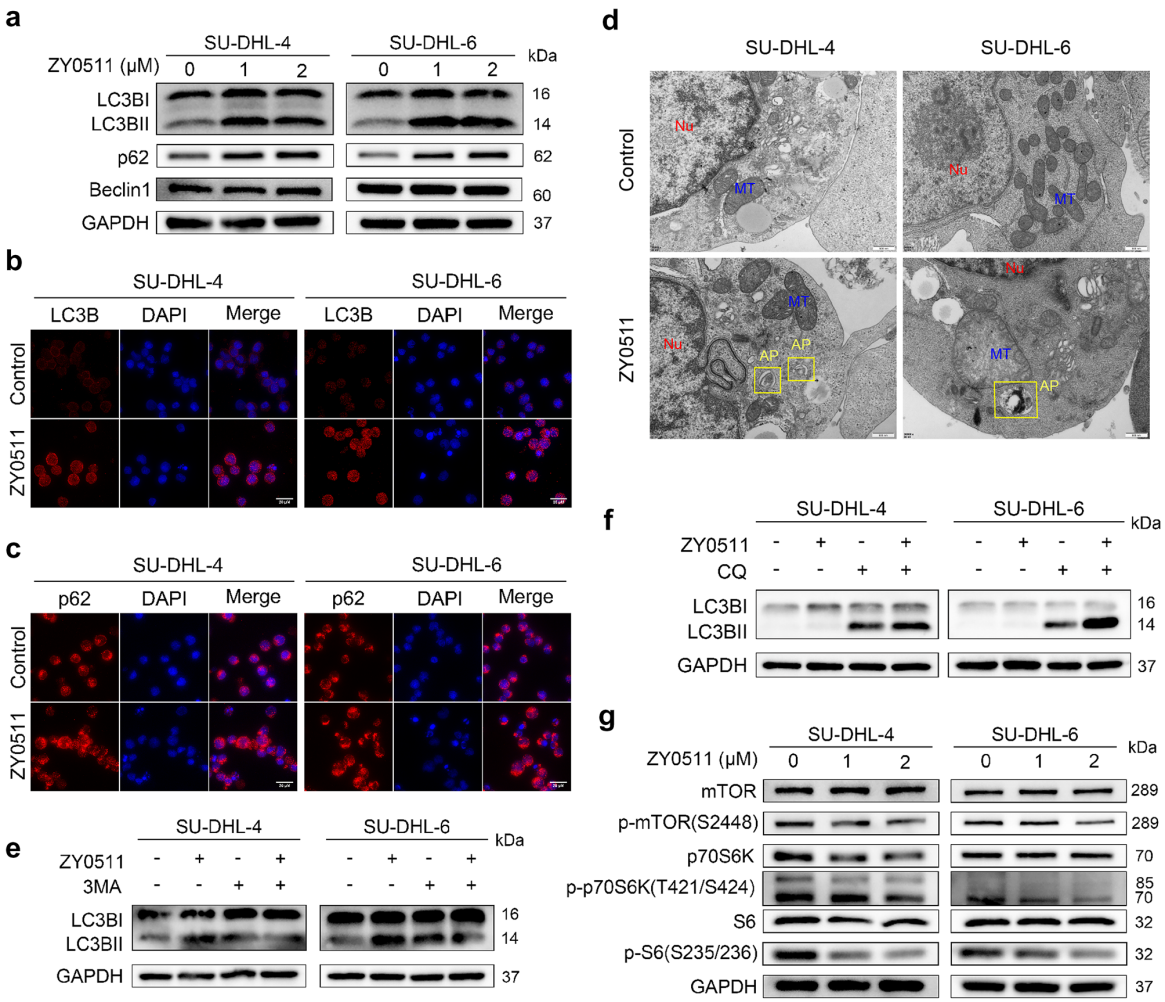
ZY0511 induces autophagy in DLBCL Cells. **a** Western blot detection of LC3B, p62, and Beclin-1 in DLBCL cells after ZY0511 treatment (*n* = 3). **b** Representative images of LC3B detected by immunofluorescence, Nuclei are labeled with DAPI. Scale bar, 20 μm. **c** Representative images of p62 detected by immunofluorescence, Nuclei are labeled with DAPI. Scale bar, 20 μm. **d** Representative TEM images of autophagosomes in DLBCL cells with ZY0511 treatment. AP, autophagosome (yellow square); M, mitochondria; Nu, nucleus. Scale bar, 500 nm. **e** Western blot detection of LC3B with ZY0511 and 3MA treatment (*n* = 3).** f** Western blot detection of LC3B with ZY0511 and CQ treatment (*n* = 3). **g** Western blot detection of the mTOR/p70S6K/S6 signaling pathway related proteins (*n* = 3)

Besides apoptosis, cell death is mediated by multiple mechanisms such as autophagy and necrosis. As RNA-seq results showed that ZY0511 induced autophagy in DLBCL cells, we detected the levels of LC3B, p62, and Beclin-1, the markers of autophagy. After 24 h treatment, ZY0511 significantly upregulated the expression of p62 and meanwhile strongly induced LC3BI converting into LC3BII, while the level of Beclin-1 remained unchanged (Fig. 5a). This result was further confirmed by immunofluorescence staining. The levels of LC3B and p62 were obviously higher in ZY0511 group than in the control group (Fig. 5b, c). Moreover, TEM detection revealed that ZY0511 treatment induced formation of autophagosomes in DLBCL cells (Fig. 5d). To further proof that autophagy is specifically induced by ZY0511, 2.5 mM 3MA or 10 μM chloroquine (CQ), autophagy inhibitors, was added into the culture media with ZY0511 [19]. As an upstream inhibitor of autophagy, 3MA treatment decreased the formation of LC3BII induced by ZY0511 treatment (Fig. 5e). As CQ is a downstream inhibitor of autophagy, it promoted the conversion of LC3BI to LC3BII when it combined with ZY0511 (Fig. 5f). In conclusion, these results confirmed that ZY0511 triggers excessive autophagy [20].

In order to clarify how ZY0511 induced autophagy, we detected the effects of ZY0511 on mTOR/p70S6K signaling pathway which play a crucial role in regulating autophagy. We found that ZY0511 reduces phosphorylation of mTOR, p70 S6 Kinase (p70S6K),and ribosomal protein S6 (S6) in a concentration-dependent manner (Fig. 5g), suggesting that autophagy induced by ZY0511 was at least in part mediated by the mTOR/p70S6K signaling pathway.

### ZY0511 inhibits tumor growth in vivo

**Fig. 6:**
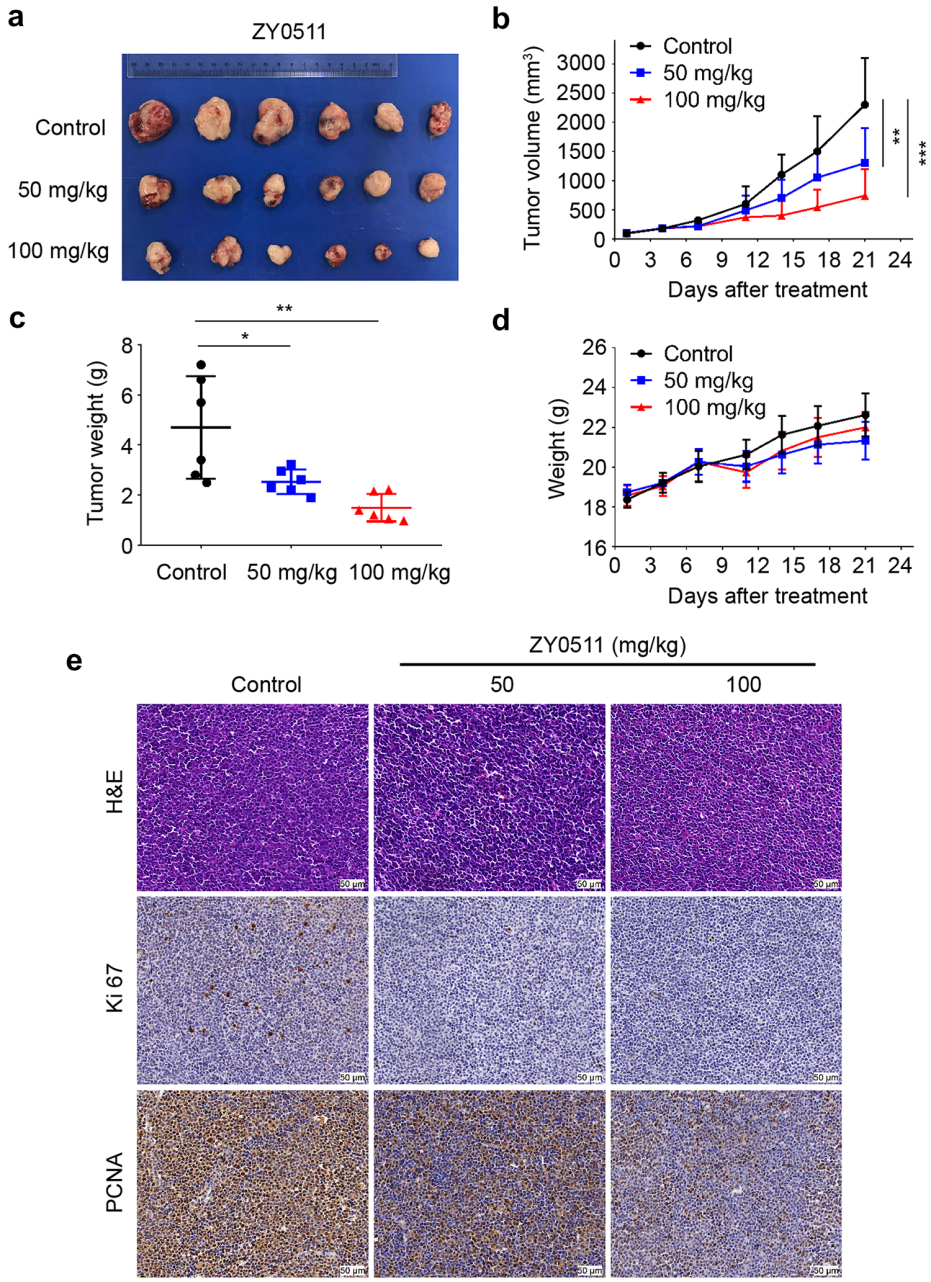
The antitumor effect of ZY0511 against DLBCL in vivo. **a**–**c** The tumor images (**a**), tumor volumes (**b**), and tumor weights (**c**) for SU-DHL-6 xenograft model treated with vehicle, ZY0511 (50 mg/kg) and ZY0511 (100 mg/kg), respectively (*n* = 6 per group). The data are presented as the mean ± SEM. *P < 0.05, **P < 0.01, one-way ANOVA followed by Dunnett’s test. **d** The body weights of SU-DHL-6 xenograft model mice during treatment with ZY0511 (*n* = 6 per group). The data are presented as the means ± SEM. **e** Representative images of the HE, Ki67, and PCNA staining of tumors after ZY0511 treatment. Scale bar, 50 μm

The above studies confirmed the in vitro anti-tumor activity of ZY0511. Next, we evaluated the in vivo anti-tumor efficacy of ZY0511 using subcutaneous xenograft models of SU-DHL-6 cells, which was the most sensitive cell line to ZY0511. NOD/SCID mice were injected intraperitoneally with ZY0511 daily for 21 days. The results showed that ZY0511 resulted in significant tumor growth inhibition with a 46% tumor-inhibitory rate at the dose of 50 mg/kg, and a 68% tumor-inhibitory rate at the dose of 100 mg/kg (Fig. 6a–c). Notably, ZY0511 did not result in significant body weight loss of mice (Fig. 6d). Consistent with the in vitro data, the Ki67 and PCNA levels in tumor tissues were markedly decreased, indicating ZY0511 treatment inhibited the growth of tumor cells in vivo (Fig. 6e).

## Discussion

As a genetically heterogeneous tumors, the clinical outcome of DLBCLs remains far from satisfactory within current clinical treatment. It is urgently needed to develop novel treatment strategies to improve the therapeutic outcomes of DLBCL. Thus, we investigated the anti-tumor effect of ZY0511 against DLBCL cells and the underlying mechanisms. ZY0511 induced the proliferation inhibition of DLBCL cells. Consistent with in vitro data, ZY0511 significantly suppressed SU-DHL-6 xenograft tumor growth in vivo. We further revealed that inhibition of LSD1 increased level of apoptosis and autophagy, which synergistically resulted the death of DLBCL cells. Collectively, our study provides a LSD1 inhibitor-based novel strategy for the clinical treatment of DLBCL.

Recently the anti-tumor effect and mechanism of epigenetic inhibitors against DLBCL attracted increasing attention. It was found that pharmacologically inhibition of CREBBP/EP300, histone acetyltransferase, resulted synthetic lethality of DLBCL cells [21]. Tazemetostat, an EZH2 inhibitor, entered phase II clinical trial for NHL treatment, including DLBCL [22]. The LSD1 inhibitors which are under clinical trial mainly focus on treatment of AML and MDS, and only one LSD1 inhibitor, CC-90011 is undergoing clinical trials for NHL therapy. To potentially use LSD1 inhibitors for DLBCL treatment, it is important to define the role of LSD1 on DLBCL progression, so that therapeutic approaches targeting this demethylase can be applied precisely. We found that *KDM1A* is significantly increased in human DLBCL tissues compared with normal tissues, and is negatively corrected with overall survival of DLBCL patients. This is not surprising as the elevated expression of LSD1 were found in various hematological malignancies such as AML. This result support our speculation that LSD1 inhibitor might be a potential epigenetic strategy for treatment of DLBCL patients.

ZY0511 is a potent LSD1 inhibitor developed by our group [12]. We found it inhibited the proliferation of various cancer cells, including hematological malignancies, cervical cancer cells, melanoma cells, and potentiated the sensitivity of 5-FU to colorectal cancer cells [23, 24]. Here, we confirmed that ZY0511 inhibited DLBCL cells growth both in vitro and in vivo [24]. Compared with ZY0511, GSK2879552 which is Tranylcypromine (TCP)-based LSD1 inhibitor at the concentration of 50 μM had no inhibitory effect on DLBCL cells (data not shown). The data are consistent with reports that TCP-based LSD1 inhibitors including GSK2879552, ORY-1001, IMG-7289 mainly inhibited the proliferation of AML and MDS, but not DLBCL and solid tumors. However, SP2509, a LSD1 inhibitor which has similar structure with ZY0511 exhibited proliferation inhibition against DLBCL cells with IC_50_ values ranging from 0.24 to 0.77 μM at 144 h (data not shown). The difference of chemical structure might be the one of reason to explain above difference. Our previous study found that ZY0511 formed hydrogen bond with Q358 of LSD1, and a π–π reaction between ZY0511 and FAD which is different from TCP derivatives. As FAD is a very important co-factor in cancer progression, inhibition of FAD by ZY0511 might be responsible for, and enhanced the anticancer effect of ZY0511 compared with TCP derivatives. However, the underlying mechanism needs to be further revealed. Moreover, the clinical trials of GSK2879552 against AML and SCLC were terminated because of adverse events and serious adverse events, suggesting that TCP derivatives may has toxicity. Thus, the further structure–activity relationship studies of ZY0511 may lead to develop safer and more potent LSD1 inhibitors which inhibits proliferation of DLBCL, and expand the clinical use of LSD1 inhibitors.

Cancer is characterized by aberrant cell cycle. Cyclin-dependent kinases (CDKs) interact with cyclin to regulate cell cycle progression and proliferation [25]. Cyclin D-CDK4/CDK6 complex promotes cells progression from G0/G1 phase to S phase, at which time DNA replication begins [26]. The genetic knockdown or pharmacological inhibition of LSD1 induces G0/G1 cell cycle arrest [26]. Similar with previous study, our results showed that ZY0511 blocked cell cycle at G0/G1 phase with the decrease of CDK4 and Cyclin D1. Moreover, we found ZY0511 increased the protein level of p53 and p–p53. The previous studies have identified that p53 is the non-histone substrate of LSD1, which prevents p53 binding to DNA by maintaining p53 in an inactive state [27]. Kuang’s group confirmed that p53–p21 pathway activation leads to CDK4 inhibition, thus resulting G1 cell cycle arrest [28]. Thus, ZY0511 may inhibit Cyclin D1 expression and CDK4 expression though the activation of p53, thus disrupt the relevant cyclin D-CDK4 complex. Besides regulated p53, it has been shown that LSD1 demethylated H3K9me2 at S-phase gene promoters (*SKP2* and *CDC25A*) and thus facilitates the expression of S-phase genes and G1-S phase transition [26]. We found that ZY0511 significantly increased the H3K9me2 levels, and decreased *SKP2* expression by 46% and *CDC25A* expression by 40%. Thus, we speculate that ZY0511 increases the level of H3K9me2 and thereby represses expression of the targeted genes of *SKP2* and *CDC25A*. However, whether ZY0511 down-regulate the expression of *CDK4* and *Cyclin D1* by directly up-regulating H3K9me2 at these genes promoter needs further study. Collectively, the cell cycle progression was blocked by ZY0511, further leading to inhibition of cell proliferation in vitro.

It is well known that cells with cell cycle arrest eventually progress to apoptosis. Apoptosis plays a crucial role in maintaining cellular homeostasis and controlling the cell proliferation [29]. There are two major apoptotic pathways, the mitochondria-mediated endogenous and exogenous apoptosis that center on the activation of caspases [30, 31]. After receiving apoptosis signal, mitochondrial protein Bax relocates to the surface of the mitochondria, resulting in membrane potential decrease and permeability increase [32]. Caspase-8 is the crucial protein in the caspase family, activated in the extrinsic apoptotic pathway, resulting in activation of caspase-3 which can cause an apoptotic cascade [33]. Once LSD1 interacts with p53, both the transcriptional activation and pro-apoptosis effects of p53 will be inhibited [27, 34]. In this study, we found that ZY0511 activated mitochondrial-mediated intrinsic apoptosis which supported by mitochondrial depolarization and pro-apoptotic protein Bax upregulation. Meanwhile, cleaved caspase-8 and cleaved caspase-3 was increased after ZY0511 treatment, suggesting that inhibition of LSD1 leads to both intrinsic and extrinsic apoptosis. These results are consistent with previous findings. Pharmacological targeting of LSD1 by pargyline or TCP induced apoptosis in oral squamous cell carcinoma cells [35]. The silencing of LSD1 also resulted apoptosis in JeKo-1 and MOLT‑4 cells [36]. Pharmacological inhibition of LSD1 up-regulates BBC3/PUMA (a pro-apoptotic protein) expression, via upregulation of H3K4me2 and downregulation of H3K27me3 [37]. Furthermore, Z-VAD-FMK, a pan-caspase inhibitor, obviously rescued ZY0511 induced apoptosis, suggesting that ZY0511 induced apoptosis plays an important role in DLBCL cell proliferation inhibition.

Together with apoptosis, autophagy involves in controlling cell fate. Autophagy is originally identified as a cell survival mechanism, sequestering, degrading, and recycling cellular material. Accumulating studies suggest that autophagy facilitate DLBCL cell death by providing a scaffold for the cell death machinery [38]. Xu’s study reported that histone lysine methyltransferase 2 inhibitor, BIX-01294, inhibits DLBCL cells proliferation by inducing endoplasmic reticulum stress-mediated autophagy [39]. Another study showed that inhibiting lncRNA MALAT-1 decreases the chemotherapy resistance of DLBCL by activating autophagy [40]. Therefore, it is feasible to mediate the death of DLBCL cells by activating autophagy. Increasing studies identified that inhibition of LSD1 by knockdown or pharmacological inhibition, mediates autophagy and contributes to cell death [41–45]. Our study showed that ZY0511 increased LC3BII/LC3BI ratio, the p62 expression, and autophagosomes formation in DLBCL cells, suggesting that ZY0511 significantly induced autophagy [24, 46]. LSD1 exerts its role by regulating the transcription of *SQSTM1* (gene encodes p62 protein) and affecting autophagy level through its H3K4 demethylase activity. Inhibition of LSD1 activates autophagy by stabilizing SQSTM1/p62, thus reduces malignant cell growth [45]. Our results revealed that ZY0511 increased *SQSTM1* expression by 1.9 folds and elevated the protein level of p62. As LSD1 inhibits the transcription of *SQSTM1* by removing the lysine residues of histone H3K4 [19], thus ZY0511 may up-regulate the expression of *SQSTM1* by up-regulating H3K4me and thereby induces autophagy. It is also reported that LSD1 inhibits autophagy in neuroblastoma by SESN2 (Sestrin2)-dependent pathway [44]. SESN2 is a downstream target gene of LSD1. It directly suppresses mTORC1 activity via interacting with GATOR2, thus results in autophagy inhibition in neuroblastoma cells [44, 47]. Our results showed that ZY0511 strongly increased *SESN2* expression, and obviously suppressed mTOR/P70S6K axis by inhibiting the level of phosphorylation of mTOR, p70S6K, and S6, which is consistent with above studies. Moreover, these data are consistent with our previous findings that ZY0511 predominately elevates the level of DDIT4 [24], a suppressor of mTORC1 pathway, and inhibits mTOR/P70S6K axis, resulting in cancer cell growth inhibition. Thus, LSD1 inhibition by ZY0511 may promote the expression of SESN2, and then inhibition mTORC1 and ultimately enhance autophagy [48, 49].

Several studies have suggested that inhibition of autophagy could reduce anti-tumor T cell responses [50], because the anti-tumor immune system recognition and response need autophagy of dying tumor cells. Shi’s group reported that inactivation of LSD1 recruits T cells to accumulate to the tumor sites [51]. As we found that ZY0511 induced autophagy, it is possible that ZY0511 may activate the recruitment of T cells into the tumor site by inhibiting LSD1 and inducing autophagy. However, substantial research is needed to further explore whether ZY0511 can stimulate anti-tumor immune response by pharmacologically inhibiting LSD1 [52].

In summary, we have demonstrated that ZY0511 showed anti-tumor effect against human DLBCL cells by blocking G0/G1 cell cycle progress and triggering apoptosis. Additionally, ZY0511 induced autophagy through mTOR/P70S6K axis. Moreover, ZY0511 inhibited tumor growth in vivo without adverse events. Therefore, our findings suggest that ZY0511 might be a promising treatment strategy for treating DLBCL.

## Supplementary Information

Below is the link to the electronic supplementary material.

Supplementary file1 (DOCX 16 kb)

Supplementary file2 (DOCX 28 kb)
