# Supplementary material for: The novel LSD1 inhibitor ZY0511 suppresses diffuse large B-cell lymphoma proliferation by inducing apoptosis and autophagy
Source: Med Oncol. 2021 Sep 7;38(10):124. doi: 10.1007/s12032-021-01572-0 (PMC8423655; doi:10.1007/s12032-021-01572-0)
Supplement: Supplementary file 1 — Supplementary file1 (DOCX 16 kb) [file 12032_2021_1572_MOESM1_ESM.docx]

Table S1. Primer sequences in RT-qPCR

| Target | Forward primer | Reverse primer |
| --- | --- | --- |
| *GAPDH* | CAGGAGGCATTGCTGATGAT | GAAGGCTGGGGCTCATTT |
| *MYC* | ATGCCCCTCAACGTTAGCTT | CTCCTCCTCGTCGCAGTAGA |
| *PCNA* | CCTGCTGGGATATTAGCTCCA | CAGCGGTAGGTGTCGAAGC |
| *CDKN1A* | AGGGGACAGCAGAGGAAGAC | GCCGTTTTCGACCCTGAGAG |
| *ATM* | TGCTTATCTGCTGCCGTCAA | TCAGGATCTCGAATCAGGCG |
| *CDK4* | GTGTACAAGGCCCGTGATCC | GTCGCCTCAGTAAAGCCACC |
| *CDK6* | CAGGTGGCCCTCGGAATAGA | ACATGACACCTACGAGGGCA |
| *CCND1* | GCCCTCGGTGTCCTACTTCAAATG | TCCTCCTCGCACTTCTGTTCCTC |
| *FAS* | TCTGGTTCTTACGTCTGTTGC | CTGTGCAGTCCCTAGCTTTCC |
| *mTOR* | GCACGTCAGCACCATCAACCTC | CTCAGCCATTCCAGCCAGTCATC |
| *BNIP3* | CAGCGTTCCAGCCTCGGTTTC | AGCTACTCCGTCCAGACTCATGC |
| *ULK1* | CTGCCTGTCGTCCACTGTGAAG | CCGCTGCTTGTCCAGGAAGAAG |
| *ATG9A* | AGCAGGTTCAGCGGGATGGAG | ACCACATTTGCGATAAGGCTCAGG |
| *SQSTM1* | TGATTGAGTCCCTCTCCCAGATGC | CCGCTCCGATGTCATAGTTCTTGG |
| *ATG7* | GAGCAAGCCCGCAGAGATGTG | TCCCAAAGCAGCATTGATGACCAG |
| *ATG3* | AGGCATACCTACCAACAGGCAAAC | CCATCCGCCATCACCATCATCTTC |
| *MAP1LC3B* | GCAGGGTAAACGGGCTGTGTG | GAGTGAGGACTTTGGGTGTGGTTC |
| *BECN1* | CCATGCAGGTGAGCTTCGT | GAATCTGCGAGAGACACCATC |
