# Supplementary material for: The novel LSD1 inhibitor ZY0511 suppresses diffuse large B-cell lymphoma proliferation by inducing apoptosis and autophagy
Source: Med Oncol. 2021 Sep 7;38(10):124. doi: 10.1007/s12032-021-01572-0 (PMC8423655; doi:10.1007/s12032-021-01572-0)
Supplement: Supplementary file 2 — Supplementary file2 (DOCX 28 kb) [file 12032_2021_1572_MOESM2_ESM.docx]

Table S2. The downregulated and upregulated representative genes list of the RNA-seq analysis

| Pathway | gene_name | Control_1_fpkm | Control_2_fpkm | Control_3_fpkm | ZY0511_1_fpkm | ZY0511_2_fpkm | ZY0511_3_fpkm |
| --- | --- | --- | --- | --- | --- | --- | --- |
| 10 upregulated genes of cell cycle | *CDKN1A* | 1.35 | 1.09 | 1.50 | 7.65 | 7.39 | 6.84 |
|  | *ATM* | 11.95 | 11.76 | 12.33 | 20.50 | 21.48 | 20.28 |
|  | *CDKN1B* | 9.86 | 9.70 | 9.78 | 14.99 | 15.58 | 15.55 |
|  | *CREBBP* | 14.67 | 14.84 | 13.57 | 22.16 | 22.33 | 22.90 |
|  | *CCNE2* | 6.95 | 7.16 | 7.68 | 10.75 | 11.39 | 10.98 |
|  | *SMAD3* | 16.10 | 16.25 | 16.34 | 23.27 | 24.48 | 22.93 |
|  | *FZR1* | 14.26 | 15.00 | 13.58 | 20.43 | 20.29 | 20.45 |
|  | *MAD1L1* | 9.16 | 9.48 | 8.85 | 12.84 | 12.90 | 12.26 |
|  | *ZBTB17* | 8.02 | 8.28 | 7.77 | 10.88 | 11.08 | 11.09 |
|  | *MDM2* | 8.84 | 8.17 | 8.92 | 11.74 | 11.28 | 11.05 |
|  | | | | | | | |
| 10 downregulated genes of cell cycle | *E2F4* | 49.55 | 49.30 | 48.59 | 20.02 | 20.76 | 21.60 |
|  | *WEE1* | 26.16 | 27.12 | 28.30 | 11.25 | 11.95 | 10.82 |
|  | *ANAPC7* | 10.96 | 12.00 | 11.84 | 4.81 | 5.00 | 4.67 |
|  | *CDK4* | 50.33 | 50.87 | 50.71 | 20.69 | 20.90 | 20.60 |
|  | *CDC25A* | 23.39 | 23.42 | 24.28 | 13.46 | 14.83 | 14.43 |
|  | *SKP2* | 22.59 | 21.09 | 20.86 | 11.47 | 10.86 | 12.23 |
|  | *MYC* | 162.50 | 150.66 | 150.30 | 45.17 | 44.15 | 44.88 |
|  | *PLK1* | 39.19 | 39.09 | 39.02 | 10.69 | 10.39 | 11.14 |
|  | *CCNB1* | 71.43 | 72.68 | 72.22 | 15.35 | 16.00 | 17.86 |
|  | *CDC20* | 93.65 | 98.57 | 92.12 | 13.68 | 14.52 | 16.79 |
|  | | | | | | | |
| 10 upregulated genes of apoptosis | *BBC3* | 1.50 | 1.53 | 1.19 | 4.51 | 4.50 | 4.28 |
|  | *JUN* | 0.51 | 0.51 | 0.30 | 3.10 | 3.29 | 2.96 |
|  | *HRK* | 3.65 | 2.89 | 2.81 | 20.16 | 20.95 | 19.92 |
|  | *CTSF* | 1.46 | 1.48 | 1.97 | 7.74 | 8.54 | 9.24 |
|  | *BIRC3* | 9.87 | 10.15 | 11.29 | 23.54 | 22.70 | 21.75 |
|  | *TRAF1* | 6.08 | 6.00 | 5.69 | 12.21 | 12.48 | 11.93 |
|  | *PIK3CA* | 7.47 | 7.43 | 6.86 | 13.61 | 13.67 | 13.20 |
|  | *TNFSF10* | 1.82 | 1.88 | 1.85 | 3.53 | 3.93 | 2.78 |
|  | *CASP7* | 2.80 | 2.61 | 2.91 | 4.64 | 4.54 | 4.75 |
|  | *FAS* | 0.84 | 1.13 | 1.26 | 1.70 | 1.57 | 1.49 |
|  | | | | | | | |
| 10 downregulated genes of apoptosis | *TNFRSF10A* | 21.83 | 20.25 | 21.07 | 12.18 | 10.22 | 10.38 |
|  | *BIRC5* | 44.07 | 41.81 | 43.91 | 21.95 | 22.32 | 23.03 |
|  | *TUBA1B* | 282.40 | 291.01 | 275.41 | 130.32 | 128.20 | 138.79 |
|  | *MAPK10* | 3.15 | 3.24 | 3.16 | 1.47 | 1.50 | 1.42 |
|  | *TUBA1C* | 43.83 | 45.40 | 43.99 | 19.67 | 18.98 | 20.24 |
|  | *CHUK* | 9.45 | 9.10 | 9.89 | 4.29 | 4.18 | 3.83 |
|  | *CTSC* | 7.40 | 7.22 | 7.84 | 2.86 | 3.20 | 2.80 |
|  | *AIFM1* | 26.00 | 29.88 | 26.85 | 10.75 | 10.23 | 11.25 |
|  | *EIF2S1* | 18.70 | 19.27 | 20.25 | 7.49 | 6.89 | 7.71 |
|  | *CYCS* | 86.12 | 87.17 | 90.70 | 31.29 | 29.60 | 28.99 |
|  | | | | | | | |
| 10 upregulated genes of autophagy | *BNIP3* | 12.52 | 12.31 | 13.07 | 95.62 | 90.47 | 91.58 |
|  | *IGF1R* | 0.55 | 0.59 | 0.57 | 3.55 | 3.47 | 3.50 |
|  | *ULK1* | 2.63 | 2.39 | 2.19 | 8.56 | 8.42 | 9.31 |
|  | *RRAS* | 3.07 | 2.59 | 3.05 | 7.76 | 7.68 | 8.13 |
|  | *CTSB* | 2.59 | 2.67 | 2.90 | 7.32 | 7.09 | 7.09 |
|  | *DDIT4* | 37.54 | 36.84 | 34.29 | 91.73 | 88.04 | 90.34 |
|  | *ATG9A* | 5.41 | 6.34 | 5.86 | 11.54 | 12.53 | 11.87 |
|  | *MAP1LC3B* | 20.19 | 19.44 | 20.63 | 39.73 | 41.90 | 41.29 |
|  | *SQSTM1* | 8.15 | 7.95 | 7.03 | 14.49 | 14.43 | 15.16 |
|  | *SESN2* | 12.11 | 11.73 | 11.87 | 15.42 | 15.91 | 15.54 |
|  | | | | | | | |
| 10 downregulated genes of autophagy | *EIF2AK4* | 5.71 | 5.61 | 5.64 | 3.03 | 2.92 | 2.70 |
|  | *MTOR* | 14.19 | 14.00 | 13.70 | 6.70 | 7.09 | 7.20 |
|  | *ATG101* | 10.04 | 10.08 | 9.21 | 4.54 | 4.67 | 4.89 |
|  | *MAPK10* | 3.15 | 3.24 | 3.16 | 1.47 | 1.50 | 1.42 |
|  | *ATG3* | 6.73 | 6.88 | 7.33 | 3.22 | 3.01 | 3.09 |
|  | *HMGB1* | 196.33 | 189.80 | 204.66 | 84.09 | 81.91 | 84.04 |
|  | *CAMKK2* | 9.54 | 10.32 | 9.67 | 3.98 | 3.92 | 4.33 |
|  | *EIF2S1* | 18.70 | 19.27 | 20.25 | 7.49 | 6.89 | 7.71 |
|  | *VAMP8* | 83.75 | 83.36 | 85.30 | 29.33 | 28.63 | 29.70 |
|  | *DEPTOR* | 6.03 | 6.34 | 6.19 | 0.58 | 0.62 | 0.54 |
